# ediblecity: an R package to model and estimate the benefits of urban agriculture

**DOI:** 10.12688/openreseurope.16054.1

**Published:** 2023-07-11

**Authors:** Josep Pueyo-Ros, Joaquim Comas, Lluís Corominas

**Affiliations:** 1 Institut Catala de Recerca de l'Aigua, Girona, Catalonia, Spain; 2 Universitat de Girona, Girona, Catalonia, Spain; 3 LEQUIA, Universitat de Girona, Girona, Catalonia, Spain

**Keywords:** edible city solutions, urban farming, urban food, nature-based solutions, Rstats, urban challenges, societal challenges, urban agriculture

## Abstract

Urban agriculture is gaining attraction to become one of the pillars of the urban ecological transition and to increase food security in an urbanized planet. However, there is a lack of systematic quantification of the benefits provided by urban agriculture solutions. In this paper, we present an R package to estimate several indicators related to benefits of urban agriculture. The goal is to provide a tool for researchers and practitioners interested in the impacts of urban agriculture. The ediblecity package provides functions to calculate 8 indicators: urban heat island, runoff prevention, green areas accessibility, NO2 sequestration, jobs created in commercial gardens, volunteers involved in community gardens, green per capita and, finally, food production. Moreover, the package also provides a function to generate scenarios with different implementations of urban agriculture. We illustrate the use of the package by comparing three scenarios in a neighborhood of Girona (Spain), which is included in the package as an example dataset. There, we compare scenarios with an increasing amount of urban agriculture solutions. The ediblecity package is open-source software. This allows other R developers to contribute to the package, providing new functionalities or improving the existing ones.

## Introduction

Urban agriculture is becoming one of the pillars of the urban ecological transition ( Säumel *et al.,* 2019). Likewise, urban agriculture might have a key role ensuring food security in an urbanized planet ( Barthel *et al.,* 2015). As a consequence, some research has paid attention on the actual or potential food production of urban agriculture ( Grafius *et al*., 2020; Richardson & Moskal, 2016). However, some others authors argued that the importance of urban agriculture does not reside in its ability to produce food but in the social benefits it provides, such as public health ( Soga *et al*., 2017) and social cohesion ( Säumel *et al.,* 2019). Moreover, other authors stated that urban agriculture can provide environmental benefits as well, such as climate regulation ( Clinton *et al*., 2018) or water runoff prevention ( Gittleman *et al*., 2017).

However, there is a lack of systematic quantification of the benefits provided by urban agriculture ( Langemeyer *et al*., 2021a). For instance, there is no clear evidence to what extent urban agriculture could contribute to reduce the urban heat island ( Lin *et al.,* 2015) or to a greener economy ( Säumel *et al.,* 2019). However, most studies have been focused on a single initiative and one benefit ( Artmann & Sartison, 2018).

Therefore, decision-makers, who are responsible of leading the urban transitions to more sustainable and resilient cities, are orphan of evidence in how to implement urban agriculture to maximize its impact on sustainability. Yet, several studies have provided some insights that can guide the implementation of urban agriculture. For instance, some models explore the rainwater harvesting potential of urban agriculture ( Gittleman *et al*., 2017; Lupia *et al*., 2017). Broader, Gómez-Villarino and Ruiz-Garcia (2021) developed guidelines to maximize ecosystem services through urban agriculture by applying adaptive design and providing a battery of indicators. And, as expected, many models have been developed to quantify food production and food security provided by urban agriculture by simulating a myriad of scenarios with different types of urban agriculture virtually implemented such as rooftop gardens, community gardens or private citizen-led gardens ( Grafius *et al*., 2020; Grewal & Grewal, 2012; Hsieh *et al.,* 2017; MacRae *et al*., 2010). Yet, a transferable model (applicable to any city) to assess simultaneously several environmental and social benefits is lacking.

Hence, our goal is to provide a tool to estimate those multiple benefits of urban agriculture that help decision-makers to strategically implement urban agriculture solutions. We developed the estimations for eight indicators measuring urban agriculture benefits and a functionality to create scenarios of urban agriculture based on the proportion of elements to be transformed to urban agriculture and on which elements will be transformed. Likewise, we packed all those functionalities in an R package called ediblecity. In the Methods, we present the interface of the package and then we illustrate the usefulness by applying the model to a neighborhood of Girona (Spain).

## Methods

### Implementation: the model under the package

All the equations and algorithms to model the benefits of urban agriculture were encapsulated in an R package ( R Core Team, 2022), using one function for each indicator and one function to create scenarios. The package was created using R version 4.2.1 ( R Core Team, 2022) in RStudio desktop v. 2022.07.02. The package structure was assisted by the package devtools ( Wickham *et al*., 2022d) following the principles in Wickham and Bryan (2022a). Likewise, the documentation of the functions was assisted by the package roxygen2 ( Wickham *et al*., 2022b). The dependencies of the package are:

- dplyr (>= 1.0.6) ( Wickham *et al*., 2022c)
- magrittr (>= 2.0.1) ( Bache & Wickham, 2022)
- sf (>=0.9) ( Pebesma, 2018a)
- stars (>= 0.5) ( Pebesma, 2022)
- rlang (>= 1.0) ( Henry & Wickham, 2022b)

***Urban representation of the city of interest.*** The ediblecity package provides eight functions to estimate eight different indicators and a function to generate scenarios. It takes, as a basis, a spatial representation of a city (or a part of a city) as a GIS layer. This representation must have one attribute indicating the land uses of the city, such as gardens, streets, rooftops, etc. Some indicators require specific information to be included in the representation. This is explained in each indicator’s section.

**Figure 1.**
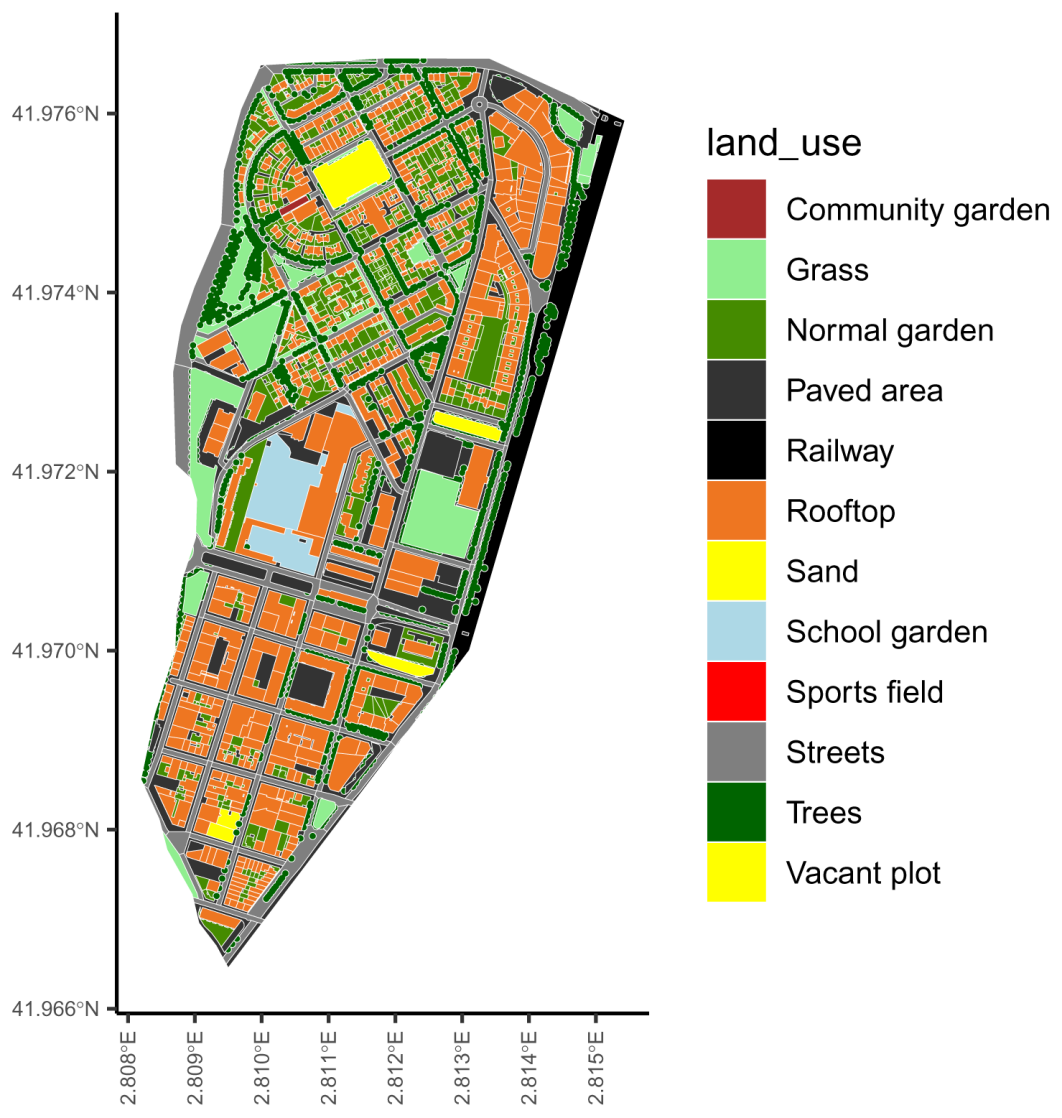
The urban representation included in the ‘ediblecity‘ package as example.

The package includes the representation of Sant Narcís, a neighbourhood of Girona (Spain) as an example of an urban representation ( Figure 1). This example can help the users to create the representation of their cities of interest. In the Table 1 below, a sample with one element of each type is shown. The representation is provided as an sf object, which is a class for spatial data in R implemented by package sf ( Pebesma, 2018b)

**Table 1.** Structure of the urban representation example.

Column	Description
land_use	A category representing the urban elements
land_use_verbose	A more detailed category for the element, for example, if it is residential
floors	The number of floors of the element, 0 for non-built
area	The surface of the element
flat_area	The surface of the element that is flat (slope < 5⍛)
edible_area	The surface that is used to grow edible plants. Only applicable to urban agriculture solutions

Along with the example for an urban representation, the ediblecity package also includes a data.frame with the default attributes of each green typology in the urban representation used to estimate the indicators ( Table 2). This includes types of urban agriculture along with other types of green infrastructure. However, the user can provide their own attributes to estimate any indicator. In city_land_uses there are other columns not shown in Table 2, they are logical variables ( *i.e.* TRUE/FALSE) used internally by the package to select urban agriculture elements.

**Table 2.** General attributes of the elements of urban green used to estimate the indicators.

land_uses	pGreen	no2_seq1	no2_seq2	food1	food2	CN1	CN2
Edible private garden	0.6	0.07	0.09	0.2	6.6	85	88
Community garden	1.0	0.07	0.09	0.2	2.2	85	88
Commercial garden	1.0	0.07	0.09	4.0	6.6	85	85
Rooftop garden	1.0	0.07	0.07	0.2	2.2	67	88
Hydroponic rooftop	1.0	0.07	0.07	9.0	19.0	98	98
Arable land	0.6	0.00	0.07	4.0	6.6	85	88
Normal garden	0.6	0.07	0.07	1.0	1.0	74	86
Permanent crops	0.6	0.09	0.09	4.0	6.6	65	77
Vacant	1.0	0.07	0.09	1.0	1.0	74	87
Grass	1.0	0.07	0.07	1.0	1.0	74	86
Mulcher	1.0	0.00	0.00	1.0	1.0	88	88
Raised bed	1.0	0.07	0.07	1.0	1.0	67	88
Trees	1.0	0.11	0.11	1.0	1.0	70	77
Vegetated pergola	1.0	0.07	0.07	1.0	1.0	98	98

pGreen is the proportion of green of the urban element. In urban agriculture solutions, this is overridden by the attribute edible_area. The following attributes come in pairs (min, max) to consider uncertainty in the estimations. The functions use a random value within the range provided by the pair of values for each element in the city. no2_seq is the capacity of the element to capture NO _2_ in gr/s. food is the food productivity in kg/m ^2^ and CN is their curve number, used to calculate infiltration rates. The details are provided in following sections.

***Indicators estimated.*** **Urban heat island** The urban heat island is a measure of how urban agriculture can contribute to climate change adaptation, specifically to adapt cities to the increasing heat waves ( Langemeyer *et al*., 2021b). The indicator uses the equation developed by Theeuwes *et al*. (2017), which was validated in 14 cities. It calculates the difference in air temperature between the urban street canyon and the rural environment. To calculate this indicator, the user must provide a raster representing the sky view factor (SVF), which describes the proportion of the unobstructed hemisphere above a certain location. SAGA, a collection of open-source algorithms for geocomputation, provides an algorithm to calculate the SVF ( Conrad, 2008).


$$UHI=\frac{1}{N}\sum_{i=1}^{N}(2\;-\;SV\;F_{i}\;-\;Fveg_{i})\;\;\times \;\;\sqrt[{4}]{\frac{\frac{Q_{q}l}{C_{\mathrm{air}}\times P_{\mathrm{air}}}\;\times \;\Delta T^{3}}{U}}$$

where *Fveg* is the proportion of vegetation in cell *i*; *Q _q_l* is the daily average global radiation (in W/m ^2^/hour); *C _air_* is the air heat capacity (in J); *P _air_* is air density (in kg/m ^3^); ∆ *T* is the difference between the maximum and minimum daily average temperatures (in ⍛C); and *U* is the daily average wind speed (in m/s).

The indicator to estimate the urban heat island is implemented by the package under the function UHI (code snippet 1). The user must provide the urban representation ( x) and the raster with SVF values ( SVF). The green_df argument is a data.frame with the proportion of green of each land_use in the urban representation ( *Fveg* in the equation). All the meteorological arguments are provided by default, based on the example provided (Mediterranean climate). However, the user can override them to provide values of their city of interest.

The function returns by default a summary of statistics of the UHI in x (min, 25%, 50%, mean, 75% and max values). If the argument return_raster is set to TRUE, the function returns a raster as stars object ( Pebesma, 2021) with the UHI values. If verbose is set to TRUE, then the function returns a vector ( *i.e.* an array) with the UHI in each cell. Both use the same resolution than SVF.

Code snippet 1: Function and arguments to estimate urban heat island.


UHI(
  x,                              # Urban representation
  SVF,                           # Raster with the sky view factor
  green_df = NULL,             # Fveg in the UHI equation
  Qql = 6.11,                   # Daily average global radiation
  Cair = 1007,                  # Air heat capacity
  Pair = 1.14,                  # Air density
  Tmax = 30.8,                  # Maximum daily average temperature
  Tmin = 20,                    # Minimum daily average temperature
  windspeed = 2.77,            # Average wind speed
  return_raster = FALSE,      # Should function return a raster or numeric values?
  verbose = FALSE              # Should the function return all the values or a summary?
)


**Runoff prevention** Surface runoff is the flow of water occurring on the ground surface when excess rainwater can no longer sufficiently rapidly infiltrate in the soil. Hence, runoff mitigation contributes to climate resilience since rain events will increase due to climate change ( Shukla *et al*., 2019). The indicator measures the runoff in the city after a specific 24-hours rain event as well as the amount of rainwater harvested by harvesting systems. We departed from the model developed by the Soil Conservation Service (USDA), known as SCS runoff curve number method ( Cronshey *et al.,* 1985).


$$Q\;=\frac{{\left(P\;-\;I_{a}\right)}^{2}}{(P\;-\;I_{a})+S}$$

where *Q* is the runoff in *mm*; *P* is the rainfall volume in mm; *I _a_* is the initial abstraction (all losses before runoff begins); and *S* is the potential soil moisture retention, which is a function of the curve number (which is determined by hydrologic soil group, see Cronshey, Roberts, and Miller (1985) for more details on the method). The SCS generalizes *I _a_* as 0 *.*2 *S*, we modified this generalization to include the rainwater harvested:


$$I_{a}\;=\;0.2S+\sum_{i=1}^{N}min{\left\{Rh,W_{s}\right\}}_{i}$$

where *Rh* is the potential water harvested by the element *i*, calculated as the amount of water fallen on the surface of adjacent higher buildings that are not used for gardening (in litres); *Ws* is the water storage capacity of the element *i* in terms of tank volume (in litres). From both, the minimum is used to calculate *I _a_*.

The runoff_prev function estimates the runoff ( *Q* in the equation) as well as the total rainfall in x and the total rainwater harvested in cubic meters (code snippet 2). Along with the urban representation (x), the user must provide a data.frame ( runoff_df argument) with four variables: land use, minimum and maximum curve numbers and a logical variable indicating whether the land use (a urban garden, a building,. . . ) has potential to harvest rainwater. The argument rain allows to set the rain event (in mm), which must be defined by the user. The curve number of each element is randomized within this range provided in runoff_df. If runoff_df is not provided, city_land_uses is used instead. Following, floors_field is the name of attribute in x that specifies the number of floors of each element; harvest_dist (in m) is the maximum distance to consider that a building is adjacent to the element; and tank_size is a range for the volume of the rainwater tank, proportional to the surface of the element (in l/m ^2^). The volume of each tank in the city is randomized within this range and multiplied by the element where they are located. All randomizations follow a random uniform distribution within the correspondent range.

Code snippet 2: Function and arguments to estimate runoff prevention


runoff_prev(
  x,                              # Urban representation
  runoff_df = NULL,             # A data.frame with land use values
  rain = 85,                     # Rain event in mm.
  floors_field = "floors",     # Variable in `x` containing the number of floors of each element
  harvest_dist = 10,            # Maximum distance to consider for rainwater harvesting surfaces
  tank_size = c(0, 45)          # Range for the size of tanks in m3
)


**Green areas accessibility** This indicator calculates the distance from each home to the closest public green area and return a summary of statistics (min, 25%, 50%, mean, 75% and max). It includes the possibility to exclude areas smaller than a threshold. The function to calculate these distances is green_distance (code snippet 3). It requires, as usual, the urban representation ( x) and a vector with all the categories considered public green areas. If it is not provided, the function uses the categories from city_land_uses where the attribute public is TRUE. The argument residence_col indicates the variable of the urban representation ( x) that must be used to identify the residences. Subsequently, residences indicates which categories of the ones contained by the variable passed to residence_col must be considered. The min_area argument can be used to exclude smaller areas than the value passed to the function (the threshold mentioned above).

If percent_out is set to TRUE, the function returns the percentage of houses that are further than max_dist argument from their closest public green area (excluding areas smaller than min_area). The default values for min_area and max_dist follow the recommendations of the World Health Organization, who recommended that all residences should be closer than 300 meters from a public green area larger than 0.5 ha.

Finally, if verbose argument is set to TRUE, a vector with all distances from residences to green areas (larger than min_area) is returned.

Code snippet 3: Function and arguments to calculate green accessibility


green_distance(
  x,                                           # The urban representation
  green_cat = NULL,                          # The land uses considered urban green
  residence_col = "land_use_verbose",     # The variable than contains information of what is a residence
  residences = "Residence",                 # The categories of former variable considered residences
  min_area = 5000,                           # Smaller areas are not included in the calculations
  percent_out = FALSE,                      # Should the function return a percentage of residences out
  max_dist = 300,                            # Which is the distaince to consider that a residence is out?
  verbose = FALSE                            # Should return a summary of distance or all values?
)


**Nitrogen dioxide sequestration** Nitrogen dioxide is a good proxy of overall air quality ( Mayer, 1999) and one of the most concerning issues in cities, with important consequences on respiratory diseases and lung cancer ( Kampa & Castanas, 2008). This indicator calculates the amount of NO _2_ sequestered by urban green and urban agriculture solutions (in g/s).


$$NO_{2}seq=\sum_{i=1}^{N}\frac{a_{i}\;\times \;cap_{i}}{1000}$$

where *a _i_* is the area (in m ^2^) of the element *i*; and *cap _i_* is the capacity of element *i* to sequester NO _2_ (in *µ*g·s ^-1^m ^-2^).

The function to estimate the NO _2_ sequestered is no2_seq (code snippet 4). It has only two arguments; the urban representation ( x) and a data.frame with four columns:

- land_uses: Column with the land use.
- no2_seq1: The low range of NO2 sequestration of each function (in *µ*g·s ^-1^m ^-2^).
- no2_seq2: The high range of NO2 sequestration of each function (in *µ*g·s ^-1^m ^-2^).
- pGreen: The proportion of green surface in each function.

When the argument is NULL (default), the function uses the city_land_uses dataset provided with the package that contains NO _2_ sequester capacity of different types of urban green.

The capacity ( *cap _i_* in the previous equation) of each element is randomized within the range provided by no2_seq1 and no2_seq2. As well the area of each element is multiplied by pGreen. In urban agriculture solutions, the attribute edible_area overrides the more general pGreen.

Code snippet 4: Function and arguments to calculate NO _2_ sequestration


no2_seq(x,                     # The urban representation
         green_df = NULL)     # The data.frame with the NO2 sequestration capacity of each land use.


**Jobs created and volunteers involved** When the urban agriculture solutions are community solutions they need volunteers to be involved. On the other hand, when they are for commercial purposes, they are supposed to create jobs. Therefore, two indicators are proposed to account for the hours of work in the ECS. One indicator relates to volunteers’ time and the other one relates to time spent by new workers (jobs created). Both indicators use the same equation:


$$jobs|volunteers=\sum_{i=1}^{N}a_{i}\;\times \;k$$

where *a _i_* is the area in m ^2^ used to grow plants ( edible_area) in the element *i*; and *k* is the number of jobs or volunteers by m ^2^. In both functions, *k* is sampled from a random uniform distribution within the specified range. Then, a Monte Carlo simulation of 1,000 iterations is executed to estimate the confidence interval. The default values were based on two sources, an empirical study ( CoDyre *et al.,* 2015) and the FoodMetres project.

The functions to calculate these indicators are edible_jobs and edible_volunteers respectively (code snippet 5). They share the same arguments, expect for jobs and volunteers, which is the value of *k* in the previous equation. As usual, the first argument is the urban representation ( x), edible is the land uses in x that are urban agriculture solutions (if NULL, city_land_uses is used as default), the attribute of x defining the area used to grow plants is area_col, the confidence interval is defined in interval, and if verbose is set to TRUE, instead of the confidence interval, the function returns a vector of length 1,000 with all the results of the Monte Carlo simulation.

Code snippet 5: Function and arguments to calculate number of jobs created and volunteers involved in urban agriculture.


edible_jobs(
  x,                                    # The urban representation
  jobs = c(0.000163, 0.022),         # The k parameter
  edible = NULL,                      # The land uses that are urban agriculture
  area_col = "edible_area",          # The variable containing the surface dedicated to grow plants
  interval = 0.95,                    # The confidence interval returned by the function
  verbose = FALSE                     # Should return all values or a summary?
)

edible_volunteers(
  x,                                    # The urban representation
  volunteers = c(0.00163, 0.22),    # The k parameter
  edible = NULL,                      # The land uses that are urban agriculture
  area_col = "edible_area",          # The variable containing the surface dedicated to grow plants
  interval = 0.95,                    # The confidence interval returned by the function
  verbose = FALSE                     # Should return all values or a summary?
)


**Green per capita** We propose an indicator to estimate green per capita at neighborhood level and at city level, including public and private gardens to account for environmental justice ( Kabisch & Haase, 2014). At the neighborhood level a ratio between the most and least green neighborhoods is calculated, with higher values meaning a major difference between neighborhoods and less spatial justice. Moreover, since wealthier areas tend to have more private gardens ( Farahani *et al.,* 2018), these can be included in the account of green per capita to not underestimate green per capita in those neighborhoods.

The function to calculate this indicator is green_capita (code snippet 6). Along with the urban representation ( x). As in other functions, the green_categories argument in the function is a list of the categories to be considered as green areas. To calculate the green per capita in the overall city, the user must provide the number of inhabitants in inhabitants. At a city level, the function returns the average amount of green per capita in the city (in m ^2^/inhabitant). Likewise there are two options to calculate green per capita at a neighbourhood level. The urban representation can contain two variables indicating the name of the neighbourhood and the inhabitants (specified in name_col and inh_col arguments respectively). Or the user can provide a GIS layer with the neighbourhoods’ boundaries and their attributes.

Furthermore, when the private argument is set to TRUE, the private gardens are also considered. Alternatively, the user can provide a list of elements to be considered as private green areas ( *e.g.* parks, urban gardens,. . . ). When the argument verbose is set to TRUE, the function returns the green per capita in each neighbourhood instead of the ratio between the most and least green ones. Finally, the argument min_inh is to exclude neighbourhoods whose number of inhabitants is under a threshold to avoid the bias in green per capita due to unpopulated neighbourhoods ( *e.g.* industrial districts).

Code snippet 6: Function and arguments to calculate green per capita.


green_capita(
  x,                            # The urban representation
  green_categories = NULL,   # The categories considered as urban green
  inhabitants = NULL,         # The number of inhabitants in the city
  neighbourhoods = NULL,      # The spatial representation of neighborhoods
  name_col = NULL,             # The variable that contains de name of the neighborhoods
  inh_col = NULL,              # The variable that contains the inhabitants of the neighborhoods
  private = FALSE,             # Should private green be considered?
  verbose = FALSE,             # Should return all the information or just the ratio?
  min_inh = 0                   # Neighborhoods with less hanbitants are excluded
)


**Food production** Although many authors stated that the main goal of urban agriculture is not to produce food ( Säumel *et al.,* 2019; Tornaghi, 2012), food production is undoubtedly an important component of urban gardens ( Furness & Gallaher, 2018; Steel, 2008) and the most frequent output modeled at a city scale ( Grafius *et al*., 2020; Grewal & Grewal, 2012). The food production is measured in terms of productivity (kg/m ^2^):


$$Food\;production=\sum_{i=1}^{N}y_{k}a_{i}$$

where *y _k_* is the yield (in *kg/m* ^2^) of the category *k* of urban garden; and *a _i_* is the area of urban garden *i* in *m* ^2^. By default, the value of *y* is randomized using a random uniform distribution within the range defined by food1 and food2 values in city_land_uses, which are the minimum and maximum yield values found in the literature for each category of urban garden. The function computes a Monte Carlo simulation of 1,000 iterations to calculate the confidence interval.

The function that calculates the food production is food_production (code snippet 7). It takes the urban representation ( x) as the first argument. If the second argument edible_df is NULL, the function uses the values from city_land_uses as specified above. Otherwise, the user can provide its own values as a data.frame with three columns:

- land_uses: specifying the category of urban agriculture, it should match the categories from x.
- food1 and food2 specifies the range of the random uniform distribution to randomize yield.

The argument area_col points to the variable of x that determines the area dedicated to grow plants in each urban garden. If NULL, the total area of each element is used instead. The number passed to interval defines which confidence interval of the food production must be returned by the function. However, if verbose is set to TRUE, the function returns a vector of length 1,000 with the results of each iteration of the Monte Carlo simulation.

Code snippet 7: Function and arguments to estimate food production


food_production(
  x,                           # The urban representation
  edible_df = NULL,          # Dataframe containing information on yields
  area_col = "edible_area", # Variable of x with surface
  interval = 0.95,           # Confidence interval returned by the function
  verbose = FALSE            # Should return all the values or just the confidence interval?
 )


***Scenarios of urban agriculture.*** The ediblecity package also provides the user with a function to create new scenarios based on the urban representation and a predefined set of urban agriculture solutions ( Table 3) based on where they are located (private gardens, plots on ground or rooftops) and their purpose (private, community or commercial). The function returns a spatial representation of the new scenario ( sf object) with the same structure of the urban representation.

**Table 3.** Elements created in new scenarios.

Urban agriculture solutions	Location	Purpose
Edible private garden	Private gardens	Private
Community garden	Plots on ground	Community
Commercial garden	Plots on ground	Community
Rooftop garden	Rooftops	Commercial
Hydroponic rooftop	Rooftops	Commercial

The location of new urban agriculture elements is randomized among all locations that fulfill the requirements of minimum area for that element. However, this is not the case for commercial gardens, they are settled in the larger available locations, assuming that commercial initiatives have the power to acquire the best spots.

The function to create a new scenario is called set_scenario (code snippet 8). It requires many arguments but most of them have default values to facilitate its use. The function needs the urban representation ( x). Then three arguments ( pGardens, pVacant, pRooftop) control the proportion of new elements that must be created. The next three arguments ( edible_area_*) control the proportion of the area of the new elements that is dedicated to grow plants ( edible_area). The edible_area of each new elements is randomized within the range provided in the arguments. The next trio of arguments ( min_area_*) specify the minimal area required to create new elements. If there are not enough elements larger than min_area_* to fulfill the first arguments, a message is displayed to inform the user (unless quiet argument is set to TRUE). Another three arguments ( *_from) control which elements can be converted from the urban representation to create new urban agriculture solutions. The argument pcommercial controls the percentage of plots on ground and rooftop that should have commercial purposes instead of community. This does not affect private gardens since they are assumed to be for personal use. Finally, area_field specifies which attribute of x must be used as the area of the elements. By default, it is am attribute called flat_area that measures the area with an slope lower than 5⍛ (in city_example).

Code snippet 8: Function and arguments to create new scenarios.


 set_scenario(
   x,                                               # The urban representation
   pGardens = 1,                                  # Proportion of private edible gardens
   pVacant = 1,                                   # Proportion of vacant plots converted to gardens on ground
   pRooftop = 1,                                  # Proportion of rooftops converted to rooftop gardens
   edible_area_garden = c(0.02, 0.3),          # Proportion of surface dedicated to grow plants in private gardens
   edible_area_vacant = c(0.52, 0.75),         # And in gardens on ground
   edible_area_rooftop = c(0.6, 0.62),         # And in rooftop gardens
   min_area_garden = 10,                         # Exclude smaller private gardens
   min_area_vacant = 100,                       # Exclude smaller vacant plots
   min_area_rooftop = 100,                      # Exclude smaller rooftops
   private_gardens_from = "Normal garden",    # Land uses to be converted to private edible gardens
   vacant_from = "Vacant",                      # Land uses to be converted to gardens on ground
   rooftop_from = "Rooftop",                   # Land uses to be converted to rooftop gardens
   pCommercial = 0,                              # Proportion of commercial gardens vs community gardens
   area_field = "flat_area",                   # Variable of x containing available surface in each element
   quiet = FALSE                                 # Should the function raise warnings?
)


### Operation

The ediblecity package is compatible with versions of R higher than 2.10. However, it was created using version 4.2.1. Code snippet 9 shows how to install the last development version, available in r-universe.

Code snippet 9: Code to install the last development version of the package


# install.packages("devtools") # if not yet installed
install.packages("ediblecity", repos = "jospueyo.r-universe.dev")


Once the package is installed, it works as any R package. It can be attached to the namespace using library(ediblecity) or preceding the functions with ediblecity::. To check the documentation of the package and its functions, type help(package="ediblecity") in the R console.

### Limitations

As all abstractions of reality, the equations and algorithms of the ediblecity package present some limitations. One limitation is the use of GIS layers to create scenarios and estimate indicators, which is intrinsically in two dimensions, and sometimes 2.5 dimensions, since we consider the height of buildings. This prevents from considering other urban agriculture solutions that are relevant such as vertical farming. To consider vertical solutions, the ediblecity package should include 3D calculations.

Another limitation is the subset of indicators chosen; this is, as we said, a choice. Other indicators might be chosen instead or added to the current subset. Hopefully, the ediblecity package will be well received by the community of R scientists and other developers will add new indicators to fulfill their own needs. Indeed, this is one of the main advantages of open-source software.

## Use cases

### Create scenarios of urban agriculture

To our understanding, the most important use case of the ediblecity package is to compare scenarios. To illustrate this, we created two scenarios and compared them with the original city_example ( Table 4). The package has been designed to work well with the tidyverse framework in R ( Wickham *et al*., 2019), especially, with the map_* family in the purrr package ( Henry & Wickham, 2022a). Therefore, we first create the two scenarios and save them in a list along with the original urban representation, which we called s0 (Code snippet 10). The scenario s1 will convert the 25% of elements to urban agriculture solutions while the scenario s2 will convert the 100%. Half of gardens in vacant plots, streets and rooftop will have commercial purposes.

**Table 4.** Number of agriculture solutions in each scenario and their surfaces (in squared meters).

land_use	s0 n	s1 n	s2 n	s0 area	s1 area	s2 area
Community garden	1	19	56	320	7024	14950
Commercial garden	0	19	56	0	28284	46769
Edible private garden	0	113	442	0	13599	55587
Hydroponic rooftop	0	76	164	0	58360	87090
Rooftop garden	0	75	164	0	14217	22485

Code snippet 10: Code to create the scenarios.



# Create new scenarios
scenarios <- map(c(0.25, 1), ~set_scenario(city_example,
                                 pGardens = .x,
                                 pVacant = .x,
                                 pRooftop = .x,
                                 private_gardens_from = "Normal garden",
                                 vacant_from = c("Vacant", "Streets"),
                                 rooftop_from = "Rooftop",
                                 pCommercial = 0.5))
#> Only 442 private gardens out of 453 assumed satisfy the 'min_area_garden'
#> Only 111 vacant plots out of 149 assumed satisfy the 'min_area_vacant'
#> Only 328 rooftops out of 604 assumed satisfy the 'min_area_rooftop'

# Add city_example as s0
scenarios[[3]] <- city_example

# Name the scenarios
names(scenarios) <- c("s1", "s2", "s0")
scenarios <- scenarios[order(names(scenarios))]


### Calculate indicators and compare scenarios

In this section, we calculate the indicators for each scenario and create tables or plots as illustration of how the results can be used.

Code snippet 11: Code to create a table with the result of urban heat island in all three scenarios.


# We use the SVF object that is provided for the city_example in the package
map_dfr(scenarios, UHI, SVF = ediblecity::SVF, .id = "Scenario") |>
  kable(caption = "Summary statistics of urban heat island effect in each scenario (in ºC)")


**Figure 2.**
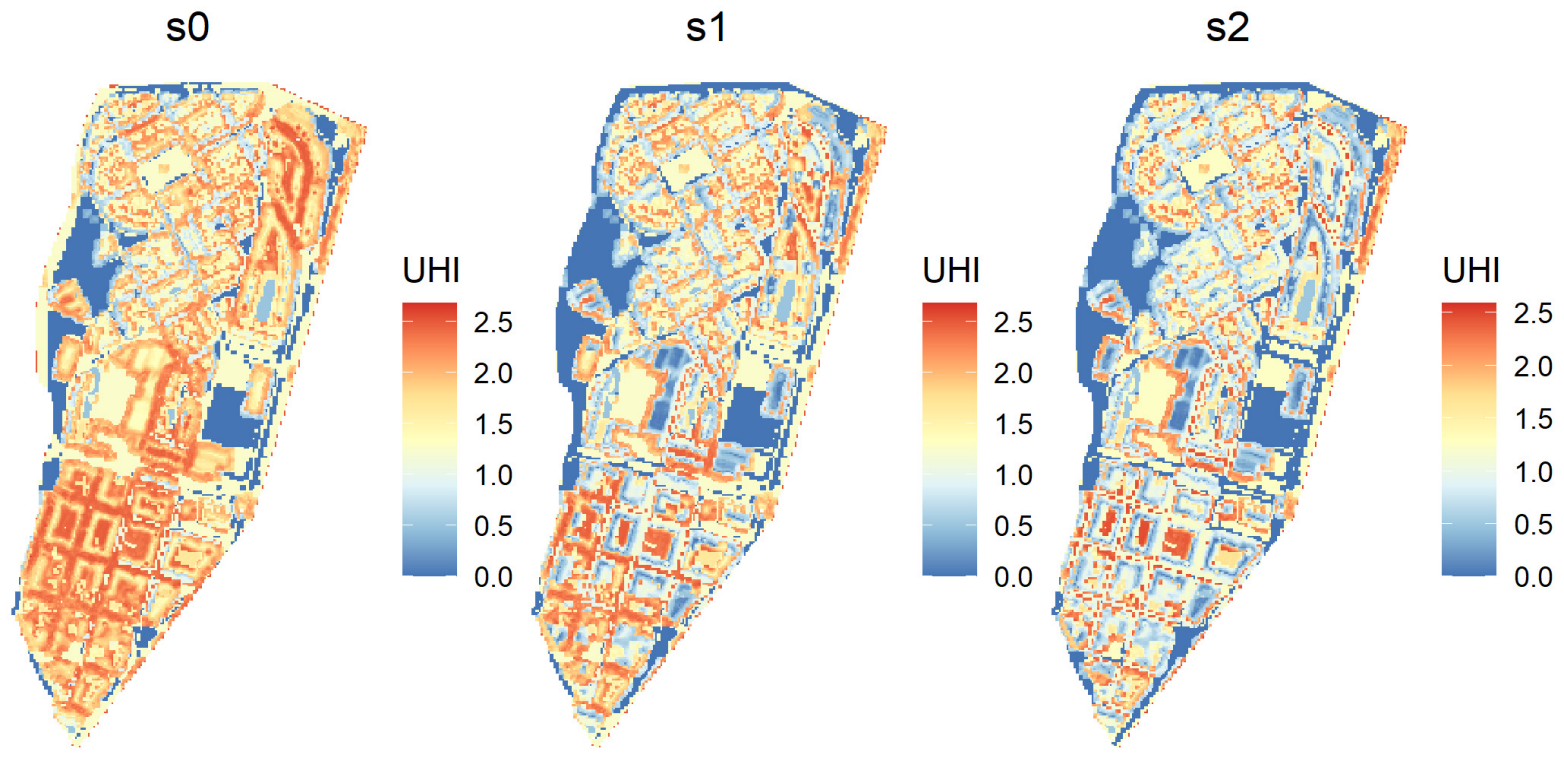
Raster returned by the UHI function when return_raster is set to TRUE.

As expected the second scenario ( s2) has the lowest values for urban heat island, but not too far from the scenario s1 ( Table 5). Both present a reduction of approximated 50% in average urban heat island regarding the base scenario. We can also generate a raster with the urban heat island for each scenario, like in Figure 2

**Table 5.** Summary statistics of urban heat island effect in each scenario (in ⍛C).

Scenario	Min.	1st Qu.	Median	Mean	3rd Qu.	Max.
s0	0	1.25	1.60	1.49	2.1	2.7
s1	0	0.64	1.25	1.18	1.9	2.7
s2	0	0.53	0.97	0.99	1.3	2.6

Code snippet 12: Code to calculate the runoff prevention in each scenario.


map_dfr(scenarios, runoff_prev, .id="scenario") |>
  kable(caption = "Runoff (mm), total rainfall (mˆ3ˆ) and rainwater harvested in each scenario (mˆ3ˆ)")


The total rainfall presented the same value in all scenarios because we used the same rain event and all scenarios represent the same total area ( Table 6). Moreover, although there was an important reduction of runoff and an increase in rainwater harvested regarding the base scenario, there was no improvement from scenario 1 to scenario 2. As shown in Code snippet 12, the rainwater harvested was larger in scenario 1. This is explained because the algorithm uses as catchment areas all adjacent upper areas that are not used for urban agriculture. Hence, as rooftop converted to urban agriculture increases, the availability of catchment areas decrease. The infiltration rates also increase because rooftop gardens retain water but this is not enough to compensate the reduction in harvesting.

**Table 6.** Runoff (mm), total rainfall (m ^3^) and rainwater harvested in each scenario (m ^3^).

Scenario	runoff	rainfall	rainharvest
s0	38	108169	1188
s1	35	108169	2040
s2	35	108169	1760

**Figure 3.**
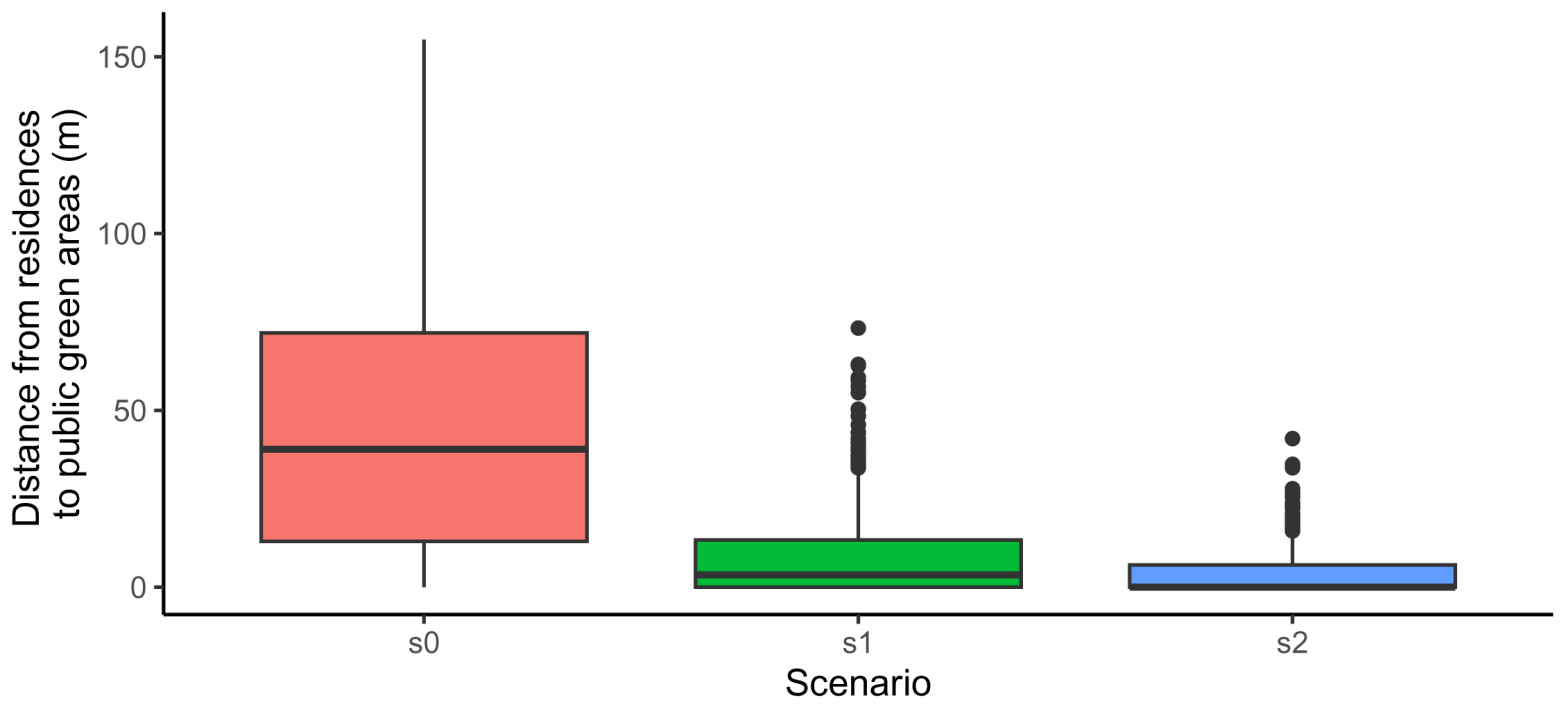
Comparison among scenarios of the distances to each residences to its closest public green area.

Code snippet 13: Code to generate a boxplot of distances to public green areas (result show in Figure 3).


map_dfc(scenarios, green_distance, min_area = 100, verbose = TRUE) |>
  pivot_longer(everything()) |>
  ggplot(aes(x=name, y=as.numeric(value), fill=name))+
  geom_boxplot(show.legend = FALSE)+
  labs(fill="Scenario", x= "Scenario", y="Distance from residences \nto public green areas (m)")


 Code snippet 14: Estimation of NO _2_ sequestration in all three scenarios


map_dfr(scenarios, no2_seq, .id="Scenario") |>
  kable(caption = "Sequestration of nitrogen dioxide in each scenario")


Regarding the capacity to absorb NO _2_, we see a significant improvement from base scenario to scenario 1 but not as larger from scenario 1 to scenario 2 ( Table 7).

**Table 7.** Sequestration of nitrogen dioxide in each scenario.

Scenario	gr/s
s0	102
s1	107
s2	110

In the Code snippet 15, there is an example to calculate jobs as well as volunteers in the different scenarios.

Code snippet 15: Code to estimate jobs and volunteers in each scenario.


jobs <- scenarios |>
  map_dfc(edible_jobs, verbose = TRUE) |>
  pivot_longer(everything(), values_to = "Jobs")

volunteers <- scenarios |>
  map_dfc(edible_volunteers, verbose = TRUE) |>
  pivot_longer(everything(), values_to = "Volunteers")

bind_cols(jobs, volunteers["Volunteers"]) |>
   pivot_longer(-name, names_to = "Indicator") |>
   ggplot(aes(x=Indicator, y=value, fill=name))+
   geom_boxplot()+
   labs(y = "People", fill="Scenario")


**Figure 4.**
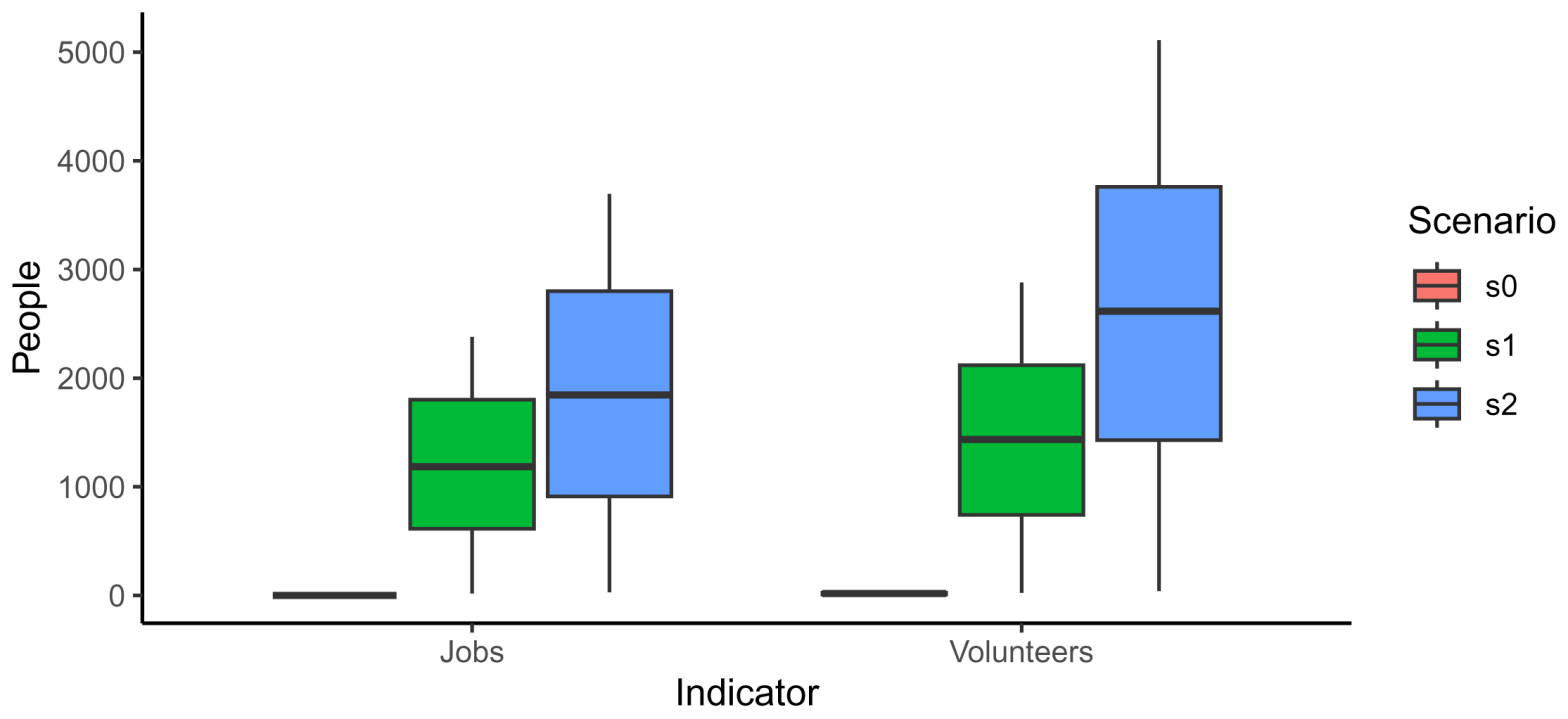
Comparison of jobs created and volunteers involved in each scenario.

As expected, the base scenario presented very low values; it created 0 jobs since there were no commercial garden and a median of 18.1 volunteers involved in one community garden. The number of jobs and volunteers also increased from scenario 1 to scenario 2 as well as the uncertainty related to the numbers ( Figure 4).

Below, we calculated the green per capita in each neighborhoods (code snippet 16). To do so, we use another spatial data set provided by the ediblecity package ( neighbourhoods_example) which contains the neighborhoods of city_example along with the inhabitants in each neighborhood.

Code snippet 16: Code to calculate the green per capita in each neighborhood


scenarios |>
  map_dfr(green_capita,
           neighbourhoods = neighbourhoods_example,
           inh_col = "inhabitants",
           name_col = "name",
           private = TRUE,
           verbose = TRUE,
           .id = "scenarios") |>
ggplot(aes(x=name, y=green_capita, fill=scenarios))+
geom_col(position = position_dodge(), color = "black")+
labs(x="Neighborhoods", y=bquote(mˆ2/person), fill="Scenario")


**Figure 5.**
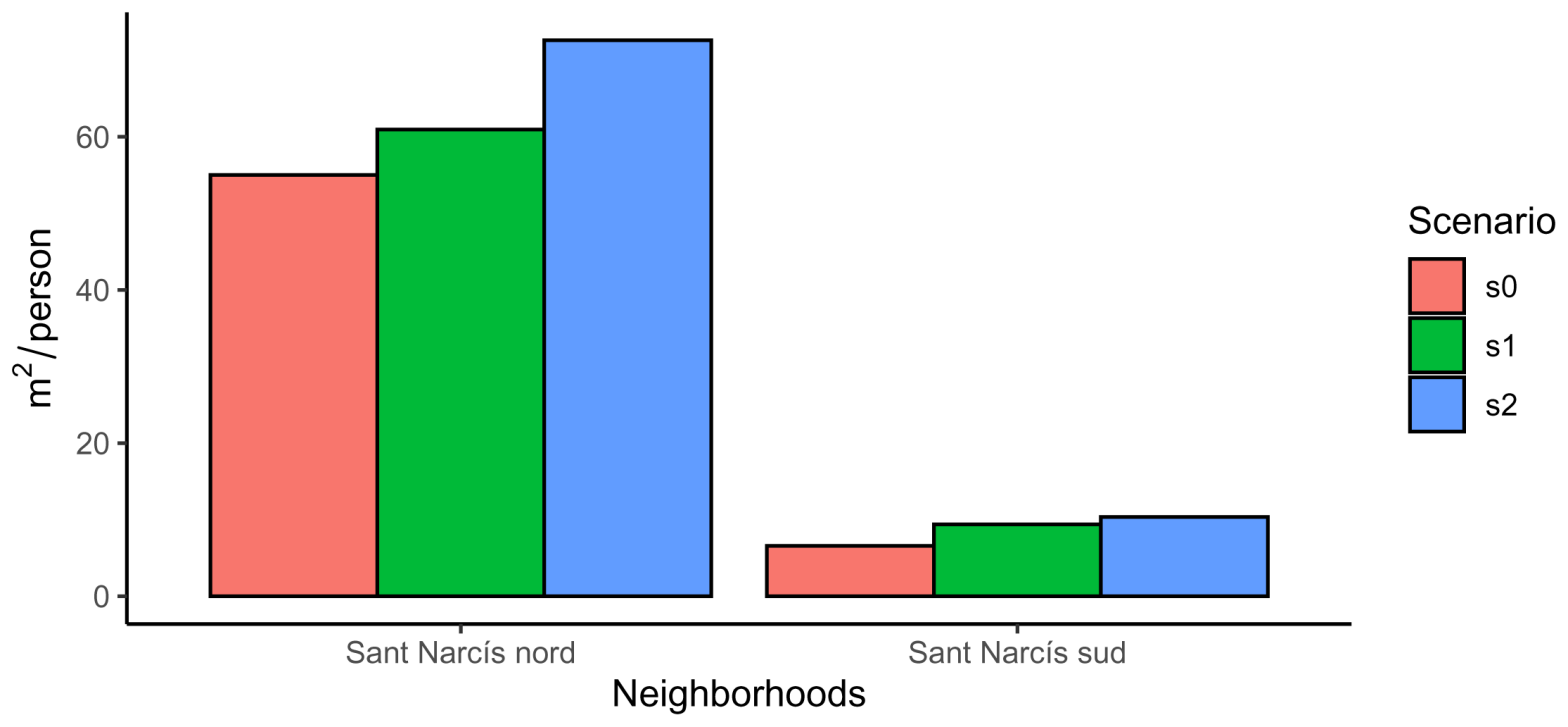
Comparison of green per capita in both neighbourhoods of Sant Narcís.

The difference between both neighborhoods ( Figure 5) is due to their urban origin. Sant Narcís nord was designed like a city garden while Sant Narcís sud is mainly composed of apartments. The interesting issue is that the improvement across scenarios is larger in Sant Narcís nord that in Sant Narcís sud, evidencing that an increase in urban agriculture is not enough to achieve environmental justice unless it is ideologically planned ( Jennings *et al.,* 2012).

The last, but not least, indicator provided by the ediblecity package is the food production. The food production is assumed to be higher in gardens for commercial purposes than in community gardens, the goal of which is not to maximize the production. This is especially the case of rooftops, since commercial rooftop gardens are assumed to use hydroponic technology while community rooftop gardens are assumed to use raised beds, following the study of Caputo, Rumble, and Schaefer (2020).

Code snippet 17: Code to get confidence intervals of food production in Tm/year.


scenarios |>
  map_dfr(food_production, .id = "Scenario") |>
  mutate(across(where(is.numeric), ~ .x/1000)) |>
  kable(caption = "Food production in Tm/year in each scenario")


Although the medians are clearly different, we cannot state that food production is bigger in scenario 2 than in scenario 1 with a 95% of confidence ( *i.e.* p-value > 0.05 in differences between s1 and s2) ( Table 8). Taking the most optimistic scenario ( s2 at quantile 95%) and considering the value per capita, the urban agriculture in our example could produce 191.94 kg/year/person. The daily intake of fruits and vegetables recommended by the FAO is 200 gr/person, *i.e.* 73 kg/person/year ( FAO & WHO, 2004). Therefore, our optimistic estimation would provide 2.63 times the neighborhood’s needs in fruits and vegetables. However, it would require (taking also the higher interval) 3,510 people working in commercial gardens and 4,829 volunteers involved in community gardens, which is 1.32 times the inhabitants of the neighborhood.

**Table 8.** Food production in Tm/year in each scenario.

Scenario	5%	50%	95%
s0	0.05	0.19	0.33
s1	457.31	610.77	780.81
s2	720.96	965.54	1215.50

### Disclaimer

Since the scenarios and the indicators have some stochastic parameters, the ideal procedure would be to integrate the creation of the scenarios and the estimation of indicators in a Monte Carlo simulation to get the confidence intervals for each combination of scenario and indicator. However, we rather kept things simple to better illustrate how to use the functions provided by the package.

## Conclusions

In this paper, we presented the ediblecity package: An R package to model and estimate the benefits of urban agriculture. The package is ready to be used by R users with a basic level. It can be used to estimate the benefits of real cases as well as to simulate scenarios. In both cases, 8 indicators are calculated. In the example illustrated in this paper, the uncertainty is captured using stochastic parameters. Moreover, the users are able to provide their own ranges in case they have more accurate data for the case study at stake. With more accurate data, the uncertainty can be easily reduced by changing the arguments of the functions. Likewise, some assumptions of the models can be overridden with truly statements.

The ediblecity package is open-source software under MIT license. This allows other R developers to contribute to the package providing new functionalities or improving the existing ones. Therefore, an open-source software is always a work-in-progress.

## Data Availability

Zenodo: icra/ediblecity: To zenodo, https://doi.org/10.5281/zenodo.7913285 Data are available under the terms of the Creative Commons Attribution 4.0 International license (CC-BY 4.0).

## Software availability

- The ediblecity package is available from https://github.com/icra/ediblecity
  Archived code at time of publication: https://doi.org/10.5281/zenodo.7913285
  License: MIT license
- This paper has been entirely written using RMarkdown, the code to reproduce the paper is available at https://github.com/icra/ediblecity_paper under MIT license.
