# Supplementary material for: ediblecity: an R package to model and estimate the benefits of urban agriculture
Source: Open Res Eur. 2023 Sep 28;3:112. Originally published 2023 Jul 11. [Version 2] doi: 10.12688/openreseurope.16054.2 (PMC10565426; doi:10.12688/openreseurope.16054.2)
Supplement: Peer review history

# Reviewer report: Reviewer response for version 2

**DOI:** 10.21956/openreseurope.17985.r35356

**Published:** 2023-10-10

**Authors:** Martí Rufí-Salís

**Affiliations:** 1 2.-0 LCA Consultants, Barcelona, Catalonia, Spain

All of my comments received a response from the authors, who either made a big change or properly defended their position. I have no further comments.

Is the rationale for developing the new software tool clearly explained?

Yes

Is the description of the software tool technically sound?

Yes

Are sufficient details of the code, methods and analysis (if applicable) provided to allow replication of the software development and its use by others?

Yes

Is sufficient information provided to allow interpretation of the expected output datasets and any results generated using the tool?

Yes

Reviewer Expertise:

Life Cycle Assessment applied to Urban Agriculture

I confirm that I have read this submission and believe that I have an appropriate level of expertise to confirm that it is of an acceptable scientific standard.

# Reviewer report: Reviewer response for version 1

**DOI:** 10.21956/openreseurope.17334.r34238

**Published:** 2023-08-24

**Authors:** Martí Rufí-Salís

**Affiliations:** 1 2.-0 LCA Consultants, Barcelona, Catalonia, Spain

**General comment**

 The authors aimed to create an R package to assess how urban agriculture and green areas interact with some indicators. The article is well-structured and concise. The chosen indicators are mostly based on published literature and formulas and are well-known to the relevant scientific community, which makes them robust. I believe that some additional justification on why the authors chose these specific indicators would further increase the robustness. In my view, this justification doesn't have to be only related to the relevancy or importance for decision-making from the selected indicators. I would definitely agree with a justification saying that this is a first shot at a wide framework that aims to increase the number of indicators in the future, either from the authors' or other contributors' side.

 Also, some additional explanation on the usability of the obtained scores would also be interesting, probably compared to potential situations. For example, the food production indicator yields a kg/m2 value, but how should this value be used? Should the reader compare it to the surrounding peri-urban/rural production? The comparison between urban agriculture neighbours and cities is well covered by the Use Cases.

**Technical comment**

 I've tried to reproduce parts of the code myself and most snippets worked using the city example created by the authors. I just encountered a problem in the code fragments where a projection was required (namely code snippet 1 - UHI and some parts in "Use Cases"). The error was the following:

*proj_create_from_database: datum not found proj_create_from_database: ellipsoid not found proj_create_from_database: prime meridian not found proj_create_from_database: datum not found proj_create_from_database: ellipsoid not found proj_create_from_database: prime meridian not found*

*Warning messages: 1: In CPL_rasterize(file, driver, st_geometry(sf), values, options, : GDAL Message 1: The definition of geographic CRS EPSG:4258 got from GeoTIFF keys is not the same as the one from the EPSG registry, which may cause issues during reprojection operations. Set GTIFF_SRS_SOURCE configuration option to EPSG to use official parameters (overriding the ones from GeoTIFF keys), or to GEOKEYS to use custom values from GeoTIFF keys and drop the EPSG code. 2: In CPL_read_gdal(as.character(x), as.character(options), as.character(driver), : GDAL Message 1: The definition of geographic CRS EPSG:4258 got from GeoTIFF keys is not the same as the one from the EPSG registry, which may cause issues during reprojection operations. Set GTIFF_SRS_SOURCE configuration option to EPSG to use official parameters (overriding the ones from GeoTIFF keys), or to GEOKEYS to use custom values from GeoTIFF keys and drop the EPSG code.*

 I have tried several ways to solve this but the problem persists. Have you faced the same problem while developing the package? I guess that if I've found this problem other people will, so maybe it will be interesting to keep a supporting file with the most common errors up to date together with the package.

 Other comments: 

- *Section Methods; Implementation: the model under the package: *It would be interesting to provide a code snippet with the package dependencies to copy-paste it into R and get the packages up and running fast.
- *Section Methods; Implementation: the model under the package: *Why only dplyr is listed as package dependency when other packages from the tidyverse package collection are used in the Use Cases section? Do you consider that Use Cases is not an explicit part of the package?
- *Statement in page 7: “The following attributes come in pairs (min, max) to consider uncertainty in the estimations. The functions use a random value within the range provided by the pair of values for each element in the city”. *Does the function pick a random value within the range considering the probability of occurrence (a value that appears more in the distribution is more likely to be picked), or just a random value (all values within the range have the same probability of being picked)?

Is the rationale for developing the new software tool clearly explained?

Yes

Is the description of the software tool technically sound?

Yes

Are sufficient details of the code, methods and analysis (if applicable) provided to allow replication of the software development and its use by others?

Yes

Is sufficient information provided to allow interpretation of the expected output datasets and any results generated using the tool?

Yes

Reviewer Expertise:

Life Cycle Assessment applied to Urban Agriculture

I confirm that I have read this submission and believe that I have an appropriate level of expertise to confirm that it is of an acceptable scientific standard, however I have significant reservations, as outlined above.

# Response

**Published:** 2023-09-18

**Authors:** Josep Pueyo-Ros

**Affiliations:** ICRA - Catalan Institute for Water Research, Spain

R3.1 I believe that some additional justification on why the authors chose these specific indicators would further increase the robustness. In my view, this justification doesn't have to be only related to the relevancy or importance for decision-making from the selected indicators. I would definitely agree with a justification saying that this is a first shot at a wide framework that aims to increase the number of indicators in the future, either from the authors' or other contributors' side.

 We explained which criteria we followed to select these 8 indicators. As well, we also explained the possibility to add new indicators by us or others: *The current version of the package includes 8 indicators related to various urban challenges defined, as defined in part by a handbook of indicators to evaluate the impact of nature-based solutions  (Dumitru & Wendling, 2021). From this source, we chose 8 indicators that meet the following criteria: (1) Measurability: The indicators can be quantitatively measured based on the area or location. (2) Direct Influence: Urban agriculture (UA) has a direct impact on the indicators, meaning that changes in UA will affect the indicator's values. (3) Relevance to UC: The indicators are directly linked to a specific UC, and at least indirectly connected to another UC, allowing us to capture multiple dimensions of urban sustainability. By considering these criteria, we ensured that the selected indicators provide a holistic understanding of the impact of UA on urban challenges.* *It's worth noting that the tool is open source. Therefore, new indicators can be added in the future either by the creators or by other users interested in specific aspects of UA.*

 R3.2 Also, some additional explanation on the usability of the obtained scores would also be interesting, probably compared to potential situations. For example, the food production indicator yields a kg/m2 value, but how should this value be used? Should the reader compare it to the surrounding peri-urban/rural production? The comparison between urban agriculture neighbours and cities is well covered by the Use Cases.

 We did not add further explanations on the interpretation of the results because we wanted to focus the attention on the usability of the tool. However, as the reviewer believes this can be useful, we added some more information on how to interpret the results.

**Urban heat island:** *The absolute reduction in degrees can also be compared to the average temperature in summer in the study area. In our case study, July 2022 had an average temperature of 28ºC. The reduction caused by UA would suppose a reduction of 3.5%.*  

**Runoff mitigation:** *However, harvested water has also some benefits, mainly water reuse. For instance, Girona’s inhabitants have an average consumption of 134 litres /day. This multiplied by the inhabitants of Sant Narcís gives a total consumption of 847 m3/day. Hence, the water harvested in scenario 1 could provide all the water in the neighbourhood during 2 days and a half.*

**Green areas accessibility:** *This indicator can be used to explore the fullfilment of WHO recommendations, which recommend that all citizens should have a green area larger than 0,5 ha closer than 300 metres (WHO, 2017).  This can be achieved by adjusting the argument min_area used to calculate the indicator that filters all the green areas smaller than the value provided to the argument.*

**NO2 sequestration:** *Considering the car emissions limitation imposed by the Euro 6 standard and assuming that cars drive at 50 km/h in the city, The sequestration of scenario 2 is approximately equivalent to neutralizing emissions from 130 cars (1.04 gr/s/car).*

**Jobs and volunteers:** *The number of jobs and volunteers can be compared to the population in the neighbourhood. In that case, using median values, the jobs of scenario 2 represents 30% of the population while the volunteers the 40%. We must note, that the scenario 2 is converting all the available streets and rooftops to urban gardens, and so the number may feel a bit overestimated.*

**Green per capita:** *However, remarkably, Sant Narcís sud increased from 6.6 to 9.4 m2/capita from base scenario to scenario 1, surpassing the standard of 9 m2/capita.* Regarding food production, we believe that the already existing explanation is sufficient: *Taking the most optimistic scenario ( s2 at quantile 95%) and considering the value per capita, the urban agriculture in our example could produce 191.94 kg/year/person. The daily intake of fruits and vegetables recommended by the FAO is 200 gr/person, i.e. 73 kg/person/year ( FAO & WHO, 2004). Therefore, our optimistic estimation would provide 2.63 times the neighborhood’s needs in fruits and vegetables. However, it would require (taking also the higher interval) 3,510 people working in commercial gardens and 4,829 volunteers involved in community gardens, which is 1.32 times the inhabitants of the neighborhood.*

 R3.3 I've tried to reproduce parts of the code myself and most snippets worked using the city example created by the authors. I just encountered a problem in the code fragments where a projection was required (namely code snippet 1 - UHI and some parts in "Use Cases"). The error was the following: *proj_create_from_database: datum not found proj_create_from_database: ellipsoid not found proj_create_from_database: prime meridian not found proj_create_from_database: datum not found proj_create_from_database: ellipsoid not found proj_create_from_database: prime meridian not found* *Warning messages: 1: In CPL_rasterize(file, driver, st_geometry(sf), values, options, : GDAL Message 1: The definition of geographic CRS EPSG:4258 got from GeoTIFF keys is not the same as the one from the EPSG registry, which may cause issues during reprojection operations. Set GTIFF_SRS_SOURCE configuration option to EPSG to use official parameters (overriding the ones from GeoTIFF keys), or to GEOKEYS to use custom values from GeoTIFF keys and drop the EPSG code. 2: In CPL_read_gdal(as.character(x), as.character(options), as.character(driver), : GDAL Message 1: The definition of geographic CRS EPSG:4258 got from GeoTIFF keys is not the same as the one from the EPSG registry, which may cause issues during reprojection operations. Set GTIFF_SRS_SOURCE configuration option to EPSG to use official parameters (overriding the ones from GeoTIFF keys), or to GEOKEYS to use custom values from GeoTIFF keys and drop the EPSG code.* I have tried several ways to solve this but the problem persists. Have you faced the same problem while developing the package? I guess that if I've found this problem other people will, so maybe it will be interesting to keep a supporting file with the most common errors up to date together with the package.

 We are aware of this issue. However, the code snippet 1 is not supposed to be evaluated, it’s meant to show the function arguments. The UHI indicator is calculated in the code snippet 11, which, in our case, raise the waring but not the error. Regarding the warning, this is very specific of our example. The problem is that spatial data in Catalonia is under the projection EPSG: 25831. The description of this projection for GeoTIFF (like SVF.tif) contains non-ANSI characters, which are not accepted by CRAN when a package is submitted. So, we replace the non-ANSI characters by the ANSI equivalents, and this is the reason of the warning. As it has no consequences in the result and it will not replicate when using a different raster, we believe is not worth to mention because it is very technical and specific to our case study. Moreover, we are still trying to find a solution.

 R3.4 Section Methods; Implementation: the model under the package: It would be interesting to provide a code snippet with the package dependencies to copy-paste it into R and get the packages up and running fast.  In principle, if the DESCRIPTION file of the package is correct, the dependencies should be installed automatically, handled by R, as happens with any package. Please, let us know if this is not the case. R3.5 Section Methods; Implementation: the model under the package: Why only dplyr is listed as package dependency when other packages from the tidyverse package collection are used in the Use Cases section? Do you consider that Use Cases is not an explicit part of the package?

 In the dependencies, we cited the packages that are dependencies of the package, but not the packages used in the Use Cases section. We agree that this should be added. We added the code snippet with the libraries used, we assume that all users will know how to install the packages if needed. We believe that including snippets with package installation is an invasion of the users’ environment. We added the following paragraph at the beginning of the section: *To replicate this section, apart from the ediblecity package, you will need to load the following packages in your R namespace using the function* *library()* *:*

- *dplyr (1.1.2) *(Wickham, François, et al., 2023)
- *ggplot2 (3.4.2) *(Wickham, 2016)
- *knitr (1.42) *(Xie, 2023)
- *purr (1.0.1) (Wickham & Henry, 2023)*
- *tidyr (1.3.0) *(Wickham, Vaughan, et al., 2023)

R3.6 Statement in page 7: “The following attributes come in pairs (min, max) to consider uncertainty in the estimations. The functions use a random value within the range provided by the pair of values for each element in the city”. Does the function pick a random value within the range considering the probability of occurrence (a value that appears more in the distribution is more likely to be picked), or just a random value (all values within the range have the same probability of being picked)?

 We could not find enough studies to draw a distribution, so we opted for a random uniform distribution, i.e., all values within the range have the same probability of being picked. We clarify this in the paper: *The following attributes come in pairs (min, max) to consider uncertainty in the estimations. The functions use a random value within the range provided by the pair of values for each element in the city. We used a random uniform distribution, i.e., all values within the rage have the same probability of being picked.*

# Reviewer report: Reviewer response for version 1

**DOI:** 10.21956/openreseurope.17334.r34240

**Published:** 2023-08-24

**Authors:** Prajal Pradhan

**Affiliations:** 1 Potsdam Institute for Climate Impact Research, Potsdam, Germany

General Comment:

 The manuscript reports an interesting software tool, i.e., an R package, which could be used to estimate the benefits of urban agriculture. Mainly, the package consists of various functions to calculate food production in urban agriculture and its benefits in terms of urban heat island effects, runoff prevention, green space, air pollution reduction, and job creation. The manuscript also presents the logic behind these calculations. The package would be interesting to promote urban agriculture. However, it does have several shortcomings. Thus, I suggest not indexing it in its current form.

 First, the manuscript could have been better embedded in the existing literature on urban gardening. There are several studies estimating the potential of urban agriculture using different scenarios and assumptions, e.g., on flat roof cover, use of roof space, urban green space, etc. [see de Simone et al. 2023 ^1^]. Further, urban agriculture also has its own limitations. For example, this systematic review ^2^ mentions health and other risks associated with urban agriculture. Thus, it would be better to embed the tool in the existing literature.

 Second, the demonstration of the applicability of the tool to other world regions is needed. This demonstration is essential for a proper justification of the use of this package. For example, it is not clear how this tool could capture variations across world regions, in terms of urban agricultural practices, crop yields, and climatic conditions. Further, I see the validation of this tool and comparison of its results with existing studies is missing.

 Third, I am wondering if the generalization of the estimated benefits based on one tool or function makes sense or not. For example, an urban heat island is a study field in itself, which is complex. It also holds for air pollution and run-offs.

 Fourth, using this package requires specific data and values for parameters. Without these data and parameters, it would not be possible for obtaining sensible results. Thus, it would be better to highlight where to get these data.

 Lastly, the manuscript is currently read like a description of the R package rather than a scientific publication. A scientific publication, here software tool article, needs to be more than the description of the R package. It requires proper embedding in the existing literature, demonstration of various cases in different world regions, validation and comparison of its results, and discussion of its findings together with limitations. Doing so would ensure greater use and value of the tool.

Is the rationale for developing the new software tool clearly explained?

Partly

Is the description of the software tool technically sound?

Partly

Are sufficient details of the code, methods and analysis (if applicable) provided to allow replication of the software development and its use by others?

Yes

Is sufficient information provided to allow interpretation of the expected output datasets and any results generated using the tool?

No

Reviewer Expertise:

urban food systems, sustainable development

I confirm that I have read this submission and believe that I have an appropriate level of expertise to confirm that it is of an acceptable scientific standard, however I have significant reservations, as outlined above.

## References

[1] : A large share of Berlin’s vegetable consumption can be produced within the city. *Sustainable Cities and Society* .2023;91: 10.1016/j.scs.2022.104362 10.1016/j.scs.2022.104362

[2] : A systematic review highlights that there are multiple benefits of urban agriculture besides food. *Global Food Security* .2023;38: 10.1016/j.gfs.2023.100700 10.1016/j.gfs.2023.100700

# Response

**Published:** 2023-09-18

**Authors:** Josep Pueyo-Ros

**Affiliations:** ICRA - Catalan Institute for Water Research, Spain

R2.1 First, the manuscript could have been better embedded in the existing literature on urban gardening. There are several studies estimating the potential of urban agriculture using different scenarios and assumptions, e.g., on flat roof cover, use of roof space, urban green space, etc. [see https://doi.org/10.1016/j.scs.2022.104362].

 We mentioned some of these papers in the introduction. For instance,  Grafius et al., (2020) or Richardson & Moskal, (2016). We also provided some references about other benefits beyond food production (Säumel et al., 2019; Soga et al., 2017). However, we decided to not enter detail with the numbers estimated on those papers for several reasons. First, because the numbers are too divergent to be reliable, for instance from a 17% from (Grewal & Grewal, 2012) to the 82% of (De Simone et al., 2023). Also, an most important, because we believe that the paper is a software tool presentation and, as such, should focus on the tool presented.  

 R2.2 Further, urban agriculture also has its own limitations. For example, this systematic review ( https://doi.org/10.1016/j.gfs.2023.100700) mentions health and other risks associated with urban agriculture. Thus, it would be better to embed the tool in the existing literature.

 We agree that urban agriculture has also some negative impacts. We chose a naïve approach and only estimated the positive impacts. This is now mentioned in the limitations section: *Likewise, we opted to only choose indicators to estimate the benefits of urban agriculture. However, urban agriculture has also some drawbacks that could be measured. Certainly, various research studies have drawn attention to a range of concerns within the realm of urban agriculture (UA). For instance, *Graefe et al. (2019) *emphasized the issue of heavy metal presence in cultivated plants, while *Perrin et al. (2015) *discussed the improper use of pesticides and fertilizers. These factors not only pose threats to both the environment and human well-being but also bring into focus the need for sustainable practices. In a similar vein, *(Whittinghill et al., 2016) *shed light on the dual nature of rooftop gardens. While they are effective in mitigating runoff, they can also contribute to nutrient runoff, especially when compared to extensive green roofs. This has implications for the quality of runoff water, underscoring the complexity of UA's environmental impacts. Moreover, beyond the potential environmental and health consequences, UA can also give rise to adverse social effects, as highlighted by *Hawes et al. (2022) *. These repercussions, like the phenomenon of green gentrification, deserve careful consideration in the context of urban agriculture development.*  

 R2.3 Second, the demonstration of the applicability of the tool to other world regions is needed. This demonstration is essential for a proper justification of the use of this package. For example, it is not clear how this tool could capture variations across world regions, in terms of urban agricultural practices, crop yields, and climatic conditions. Further, I see the validation of this tool and comparison of its results with existing studies is missing.

 We are confident that we developed a tool that is applicable to any context, because all parameters of the model can be provided by the user (yields, meteorological data, urban representation…). Therefore, there is no point to validate the model in this paper since each application of the tool should be validated on the specific case study. Validating the results of the Use case section is out of the scope of this paper, because this is only an example on how to use the tool (as explained in the guidelines of the journal). We added this clarification to the text: *While the package offers default values for nearly all necessary parameters, it's worth noting that these defaults have been tailored to our specific example, which focuses on a mid-sized Western Mediterranean European city. Nevertheless, it's important to emphasize that users have the flexibility to customize all parameters according to their specific context.*  

 R2.4 Third, I am wondering if the generalization of the estimated benefits based on one tool or function makes sense or not. For example, an urban heat island is a study field in itself, which is complex. It also holds for air pollution and run-offs.

 We totally agree with this. Our model, as all models, suffers a trade-off between simplicity and accuracy. We tried to provide a tool to estimate different benefits with the minimal information required to the user and using state-of-the-art equations and algorithms. However, this will never be more accurate than a field study with experimental measurements. As it is said, all models are wrong, but some are useful. We hope ours is useful to do a first approximation to the potential impacts of urban agriculture.

 R2.5 Fourth, using this package requires specific data and values for parameters. Without these data and parameters, it would not be possible for obtaining sensible results. Thus, it would be better to highlight where to get these data.

 We could specify where we get the data used for our example. But we believe that this is irrelevant since each case study must use the corresponding data sources. We used data from Catalan institutions such as the Catalan Cartographic Institute, the Catalan Statistics Institute and the Spanish cadastre. However, this is useless for a user trying to apply the tool in a case study outside Catalonia.

 R2.6 Lastly, the manuscript is currently read like a description of the R package rather than a scientific publication. A scientific publication, here software tool article, needs to be more than the description of the R package. It requires proper embedding in the existing literature, demonstration of various cases in different world regions, validation and comparison of its results, and discussion of its findings together with limitations. Doing so would ensure greater use and value of the tool.

 We hope that the previous comments will satisfy your concerns about the soundness of the paper. As we said, we prefer to focus on the tool and its usability rather than in the results, that are specifics of our example and not transferable at all to other case studies.

# Reviewer report: Reviewer response for version 1

**DOI:** 10.21956/openreseurope.17334.r33637

**Published:** 2023-07-26

**Authors:** Carlos Tapia

**Affiliations:** 1 Nordregio, Stockholm, Sweden

The authors provide a software tool to evaluate a selection of potential benefits of urban agriculture, focusing on the physical characteristics of the gardens. Each indicator is enabled through a devoted function with specific data requirements. The description of the computations and underlying assumptions of each function is sufficiently detailed and anchored in validated methodologies. The scenario development module looks like a decision intelligence algorithm rather than as a tool providing scenarios in which the qualities of the different agriculture developments could be compared against the indicators calculated in the previous module. The tool is deployed as an open source software package for R that users can download and use free of charge. As such, the package provides a valuable resource for city planners and urban developers engaged in the design or identification of spaces to accommodate urban gardens.

 The general relevance of the tool is well explained. In their reasoning, authors place the emphasis on the lack of tools – how to measure? –, which is justified considering the applied focus of the study. However, for the sake of completeness and consistency, it would be advisable to mention the lack of an established conceptual framework in the first place – what to measure? –. In particular, the review of potential benefits – and also the risks – linked to urban agriculture is rather thin. Authors are encouraged to add references to existing reviews about this topic, including bibliometric information. Also, authors should elaborate more on the interpretation of results: which could be the optimal/reasonable thresholds to for the different indicators?

 Authors are also suggested to expand the Limitations section to give more details about how the indicators were selected. The value ‘choice’ aspect mentioned does not seem to be enough justification. Some of the indicators related to soil coverage and distance metrics, like UHI, runoff, accessibility to gardens, green per capita, etc., seem unspecific for urban agriculture and refer to green areas in general. This should be acknowledged in the text, in particular considering the lack of more specific social indicators capturing social benefits of urban farming beyond jobs.

 Another important limitation worth mentioning refers to the transferability challenge. The great diversity of urban settings undermines the capacity of this and other planning tools to be transferred to different contexts. In particular, there is a planning divide between cities in Western countries and the Global South. Authors are encouraged to mention this limitation and explain how it could be partially addressed through parametrisation. Another relevant factor potentially limiting transferability of the tool are IT-related aspects. The R system has become increasingly user-friendly over time. Still, it remains a software development tool requiring programming skills with a steep learning curve. This may limit the use of R-based libraries by unskilled persons.

Is the rationale for developing the new software tool clearly explained?

Partly

Is the description of the software tool technically sound?

Yes

Are sufficient details of the code, methods and analysis (if applicable) provided to allow replication of the software development and its use by others?

Yes

Is sufficient information provided to allow interpretation of the expected output datasets and any results generated using the tool?

Partly

Reviewer Expertise:

Indicator-based assessments, social sustainability, social impacts

I confirm that I have read this submission and believe that I have an appropriate level of expertise to confirm that it is of an acceptable scientific standard, however I have significant reservations, as outlined above.

# Response

**Published:** 2023-09-18

**Authors:** Josep Pueyo-Ros

**Affiliations:** ICRA - Catalan Institute for Water Research, Spain

R1.1 The general relevance of the tool is well explained. In their reasoning, authors place the emphasis on the lack of tools – how to measure? –, which is justified considering the applied focus of the study. However, for the sake of completeness and consistency, it would be advisable to mention the lack of an established conceptual framework in the first place – what to measure? –. In particular, the review of potential benefits – and also the risks – linked to urban agriculture is rather thin. Authors are encouraged to add references to existing reviews about this topic, including bibliometric information.

 We mentioned some of these papers in the introduction. For instance,  Grafius et al., (2020) or Richardson & Moskal, (2016). We also provided some references about other benefits beyond food production (Säumel et al., 2019; Soga et al., 2017). However, we decided to not enter detail with the numbers estimated on those papers for several reasons. First, because the numbers are too divergent to be reliable, for instance from a 17% from (Grewal & Grewal, 2012) to the 82% of (De Simone et al., 2023). Also, and most important, because we believe that the paper is a software tool presentation and, as such, should focus on the tool presented.

 R1.2 Also, authors should elaborate more on the interpretation of results: which could be the optimal/reasonable thresholds to for the different indicators?

 We did not add further explanations on the interpretation of the results because we wanted to focus the attention on the usability of the tool. However, as the reviewer believes this can be useful, we added some more information on how to interpret the results as well as some thresholds where those existed. However, we must note that thresholds are usual arbitrary.

**Urban heat island:** *The absolute reduction in degrees can also be compared to the average temperature in summer in the study area. In our case study, July 2022 had an average temperature of 28ºC. The reduction caused by UA would suppose a reduction of 3.5%.*  

**Runoff mitigation:** *However, harvested water has also some benefits, mainly water reuse. For instance, Girona’s inhabitants have an average consumption of 134 litres /day. This multiplied by the inhabitants of Sant Narcís gives a total consumption of 847 m3/day. Hence, the water harvested in scenario 1 could provide all the water in the neighbourhood during 2 days and a half.*

**Green areas accessibility:** *This indicator can be used to explore the fullfilment of WHO recommendations, which recommend that all citizens should have a green area larger than 0,5 ha closer than 300 metres (WHO, 2017).  This can be achieved by adjusting the argument min_area used to calculate the indicator that filters all the green areas smaller than the value provided to the argument.*

**NO2 sequestration:** *Considering the car emissions limitation imposed by the Euro 6 standard and assuming that cars drive at 50 km/h in the city, The sequestration of scenario 2 is approximately equivalent to neutralizing emissions from 130 cars (1.04 gr/s/car).*

**Jobs and volunteers:** *The number of jobs and volunteers can be compared to the population in the neighbourhood. In that case, using median values, the jobs of scenario 2 represents 30% of the population while the volunteers the 40%. We must note, that the scenario 2 is converting all the available streets and rooftops to urban gardens, and so the number may feel a bit overestimated.*

**Green per capita:** *However, remarkably, Sant Narcís sud increased from 6.6 to 9.4 m2/capita from base scenario to scenario 1, surpassing the standard of 9 m2/capita.* Regarding food production, we believe that the already existing explanation is sufficient: *Taking the most optimistic scenario ( s2 at quantile 95%) and considering the value per capita, the urban agriculture in our example could produce 191.94 kg/year/person. The daily intake of fruits and vegetables recommended by the FAO is 200 gr/person, i.e. 73 kg/person/year ( FAO & WHO, 2004). Therefore, our optimistic estimation would provide 2.63 times the neighborhood’s needs in fruits and vegetables. However, it would require (taking also the higher interval) 3,510 people working in commercial gardens and 4,829 volunteers involved in community gardens, which is 1.32 times the inhabitants of the neighborhood.*  

 R1.3 Authors are also suggested to expand the Limitations section to give more details about how the indicators were selected. The value ‘choice’ aspect mentioned does not seem to be enough justification. Some of the indicators related to soil coverage and distance metrics, like UHI, runoff, accessibility to gardens, green per capita, etc., seem unspecific for urban agriculture and refer to green areas in general. This should be acknowledged in the text, in particular considering the lack of more specific social indicators capturing social benefits of urban farming beyond jobs.

 We explained which criteria we followed to select these 8 indicators. We did not include more social indicators because we developed a static spatial model, so we were limited mainly by the criteria 1. Moreover, we also explained the possibility to add new indicators by us or others: *The current version of the package includes 8 indicators related to various urban challenges defined, as defined in part by a handbook of indicators to evaluate the impact of nature-based solutions (Dumitru & Wendling, 2021). From this source, we chose 8 indicators that meet the following criteria: (1) Measurability: The indicators can be quantitatively measured based on the area or location. (2) Direct Influence: Urban agriculture (UA) has a direct impact on the indicators, meaning that changes in UA will affect the indicator's values. (3) Relevance to UC: The indicators are directly linked to a specific UC, and at least indirectly connected to another UC, allowing us to capture multiple dimensions of urban sustainability. By considering these criteria, we ensured that the selected indicators provide a holistic understanding of the impact of UA on urban challenges.* *It's worth noting that the tool is open source. Therefore, new indicators can be added in the future either by the creators or by other users interested in specific aspects of UA.*  

 R1.4 Another important limitation worth mentioning refers to the transferability challenge. The great diversity of urban settings undermines the capacity of this and other planning tools to be transferred to different contexts. In particular, there is a planning divide between cities in Western countries and the Global South. Authors are encouraged to mention this limitation and explain how it could be partially addressed through parametrisation.

 We are confident that we developed a tool that is applicable to any context, because all parameters of the model can be provided by the user (yields, meteorological data, urban representation…). We added this clarification to the text: *While the package offers default values for nearly all necessary parameters, it's worth noting that these defaults have been tailored to our specific example, which focuses on a mid-sized Western Mediterranean European city. Nevertheless, it's important to emphasize that users have the flexibility to customize all parameters according to their specific context.*  

 R1.5 Another relevant factor potentially limiting transferability of the tool are IT-related aspects. The R system has become increasingly user-friendly over time. Still, it remains a software development tool requiring programming skills with a steep learning curve. This may limit the use of R-based libraries by unskilled persons.

 This is totally true. Currently, the tool is reserved for users with programming skills. We chose R and not other languages such as Python, because we believe that R is the programming language of non-programmers. It is used by many scientific disciplines such as biology, sociology, geography… Yet, we are planning to develop a web-based graphical interface for the tool to reach a wider audience. Any suggestions for funding are welcome.
